# AI-Based Detection of Optical Microscopic Images of *Pseudomonas aeruginosa* in Planktonic and Biofilm States

**DOI:** 10.3390/info16040309

**Published:** 2025-04-14

**Authors:** Bidisha Sengupta, Mousa Alrubayan, Manideep Kolla, Yibin Wang, Esther Mallet, Angel Torres, Ravyn Solis, Haifeng Wang, Prabhakar Pradhan

**Affiliations:** 1 Department of Chemistry & Biochemistry, Stephen F. Austin State University, Nacogdoches, TX 75962, USA; 2 Department of Physics and Astronomy, Mississippi State University, Mississippi State, MS 39762, USA; 3 Department of Computer Science and Engineering, Mississippi State University, Mississippi State, MS 39762, USA; 4 Department of Industrial and Systems Engineering, Mississippi State University, Mississippi State, MS 39762, USA

**Keywords:** brightfield imaging, deep learning, aptamer DNA, silver nanocluster (Ag-NC), U-Net with ResNet, convolutional neural networks (CNN)

## Abstract

Biofilms are resistant microbial cell aggregates that pose risks to the health and food industries and produce environmental contamination. The accurate and efficient detection and prevention of biofilms are challenging and demand interdisciplinary approaches. This multidisciplinary research reports the application of a deep learning-based artificial intelligence (AI) model for detecting biofilms produced by *Pseudomonas aeruginosa* with high accuracy. Aptamer DNA-templated silver nanocluster (Ag-NC) was used to prevent biofilm formation, which produced images of the planktonic states of the bacteria. Large-volume bright-field images of bacterial biofilms were used to design the AI model. In particular, we used U-Net with ResNet encoder enhancement to segment biofilm images for AI analysis. Different degrees of biofilm structures can be efficiently detected using ResNet18 and ResNet34 backbones. The potential applications of this technique are also discussed.

## 1. Introduction

Biofilm-forming microbes synthesize different signaling biomolecules such as proteins, carbohydrates, and DNA, which help in their cooperative activities and attachments to biotic or abiotic surfaces to create organized multicellular communities called biofilm [1–3]. Studies on biofilm by the *gram*-positive bacterium were pioneered by Ferdinand Cohn in 1877 [1]. Numerous studies on the biofilms of various *Bacillus* species (*Bacillus cereus, Bacillus anthracis*, and *Bacillus thuringiensis*, *BT*) [4–7] and other bacterial species (*gram*-negative *Pseudomonas aeruginosa*, *PA*) were conducted in the last decade to understand the mechanism of the growth and control of biofilms [8,9]. Many medically important fungi form biofilms [10–12] (including yeast *Candida* [13] and mold [10], which are resistant to antifungal drugs). Biofilm formation causes chronic infections and a loss of immune responses in individuals with underlying health problems and can even lead to death, creating a severe danger to public health [14–16]. This is due to the resistance of the biofilms against antimicrobial agents [14]. Biofilms also cause significant problems by contaminating medical devices, food, and the environment [17,18], threatening healthcare industries, including hospital devices, human health, and biotech [19,20] companies. According to a study by the NIH [21], biofilm formation can be attributed to a significant portion (65% and 80%) of all microbial and related recurring infections.

Designing effective strategies to combat biofilm-related infections is crucial, as it demands a detailed understanding of the underlying processes in biofilm formation, and the unraveling their scientific implications. Phenotypes of biofilm-forming microbes differ from their planktonic states, leading to the rise of antibiotic resistance and the failure of modern antibiotics to treat biofilm infections [14,22]. An alternative approach to combating these deadly biofilms needs to be addressed.

Nanotechnology has become prominent in reducing and controlling biofilm formation [23–26]. In personal and medical care and household products, silver is one of the most widely used metals [27–29]. Recently, Ag nanoparticles (NP) were used to prevent biofilm in *gram*-positive and *gram*-negative bacteria [28,30–32]. Small DNA stretches are designed to make aptamer molecular beacons, called functionalized aptamers [33–35], for detecting biofilms. Sengupta’s group has pioneered the usefulness of DNA aptamers to scaffold and carry silver nanoclusters (Ag-NC) to influence biofilm formation [7,8]. They have also shown that a capping agent like methyl-beta-cyclodextrin (CDx) [7]-bound Ag-NC has a better preventive effect on biofilm.

The full and partial formation of biofilms by a controlling substance, by Ag-NC, is difficult to predict by a simple statistical analysis. Recently, artificial intelligence (AI approaches such as convolutional neural networks (CNN) have come into prominence for biofilm (object) detection [36–39]. The effective AI model Unet [37] is widely recognized as suitable and has the potential for different types of biomedical image segmentation. The network architecture contains a fully convolutional encoder-decoder with skip connections between the encoder blocks and their symmetric decoder blocks. ResNet [37,40] introduces the concept of skip connections, allowing the network to skip specific layers to let the model learn effectively. It can be shown that a U-Net framework built with a ResNet encoder can leverage the power of deep residual learning and enhance the feature extraction process to improve the overall segmentation performance [41]. Beyond U-Net-based approaches, other deep learning models have also demonstrated strong segmentation capabilities. DeepLabV3 uses atrous spatial pyramid pooling (ASPP) to capture multi-scale contextual information, improving segmentation accuracy for objects of different sizes [42]. Attention U-Net enhances the standard U-Net by integrating attention mechanisms, helping the model focus on important regions while reducing background noise. BASNet (Boundary-Aware Segmentation Network) prioritizes boundary refinement, making it particularly effective for applications requiring precise edge detection [43]. F-CN (Image Segmentation using Composable Fully-Convolutional Networks), a deeper fully convolutional network, efficiently learns spatial hierarchies without requiring explicit region proposals [44], though it may struggle with boundary precision. Each of these models has distinct strengths and different parameters based on the structure. The identification of biofilm is difficult for humans to identify with visual information; extensive and essential features related to biofilm are expected to be captured through a deep network learning process. Biofilm bright field images can be characterized using U-Net architecture with ResNet18 and ResNet34 backbone and other convolutional models. This research exploits an AI-based model to predict and detect the microbial biofilm of *gram*-negative *Pseudomonas aeruginosa*, PA [8] with higher accuracy against a given aptamer-DNA-based Ag-NC. Our results show that this AI model can be applied to any image to detect biofilm formation with higher accuracy.

This paper reports the prevention of PA biofilm by the Ag-NC, which is synthesized on a DNA aptamer matrix. In particular, we created biofilm using PA in 2D fully or partially on a glass slide, with or without the treatment of PA targeted DNA aptamer-enclosed Ag-NC. Large-volume bright-field images of the PA biofilms grown on glass slides were then generated using an automated transmission microscope. Finally, we analyze the large-volume bright field data via AI, especially U-net with ResNet. For the AI analyses, the AI algorithm is first fed with bright-field images with marked biofilms; then, unknown biofilms are fed to the leaned AI model for detection. The results show the detection of biofilm formation/prevention with higher accuracy.

## 2. Results and Discussions

**Figure 1.**
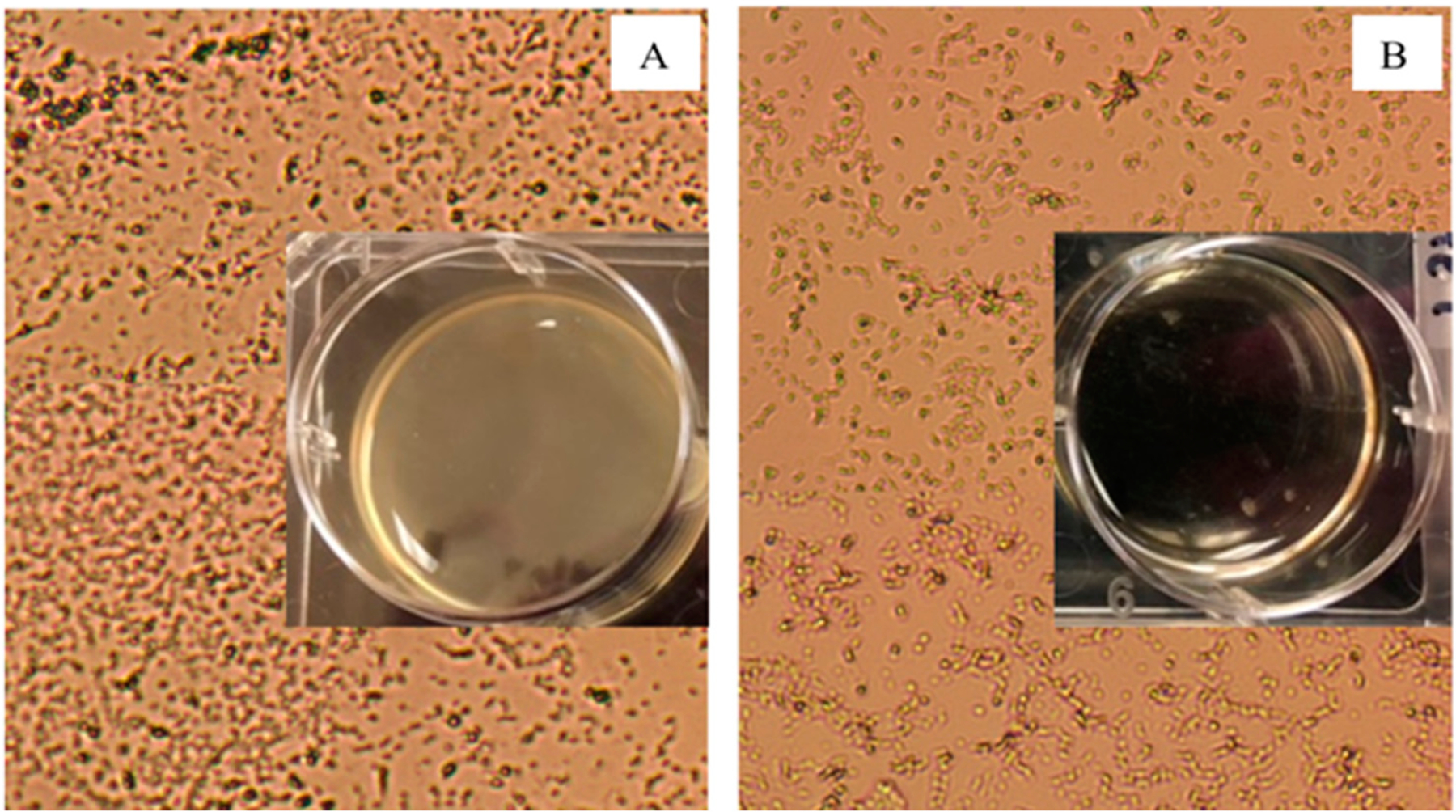
Brightfield images of *Pseudomonas aeruginosa* (PA) in Tryptic Soy Broth culture medium in the absence (control (**A**)) and presence of aptamer-DNA templated silver nanoclusters (**B**). The biofilm formation is evident by the turbidity in the control state (1A inset), while planktonic cells are observed in the Ag-NC sample (1B inset). Turbidity in well A containing control PA solution proves biofilm formation, which is cleared in the presence of Ag-NC (well B). Both the wells were photographed on a black background.

Figure 1 shows representative bright field images of control (Figure 1A) and aptamer-enclosed nanocluster-treated (Figure 1B) samples of *Pseudomonas aeruginosa*. The incubation of the PA cells with the aptamer-enclosed Ag-NC for 48 h cleared the turbidity (see Figure 1B inset), which was observed in control and was indicative of biofilm formation [7,8] (as shown in Figure 1A).

### Machine Learning Results for Biofilm Segmentation

The AI frameworks′ training inputs are image data with their corresponding biofilm annotations, and the output is the model-predicted biofilm mask. The binary cross entropy loss between the actual label and the prediction is computed and minimized in the training. The Adam optimizer with weight decay is determined with a learning rate of 5 × 10^−4^. All the models use a batch size of 8.

**Figure 2.**
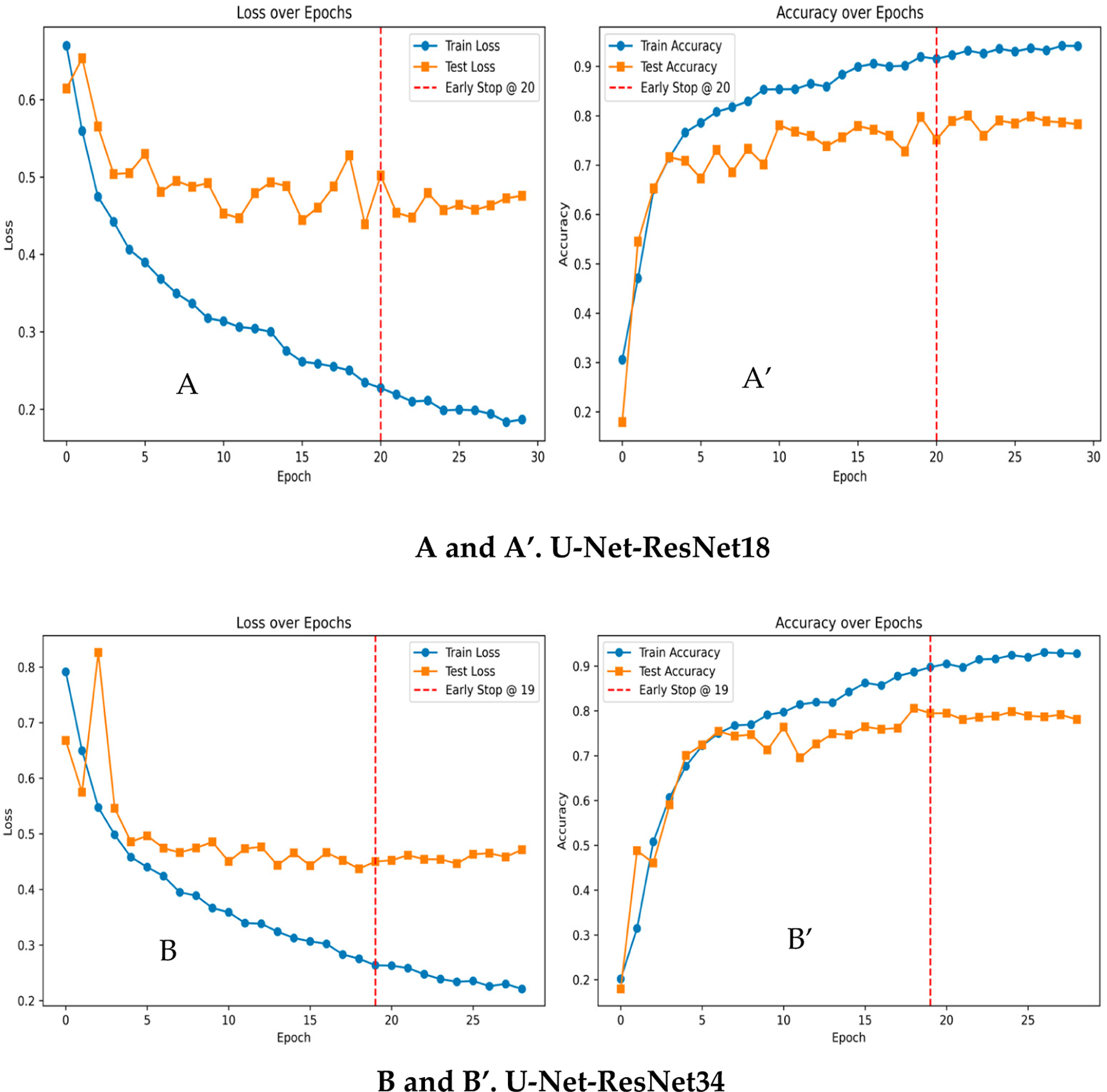
A comparison of loss and accuracy curves between the segmentation model with different backbone frameworks. (**A**,**A’**): U-Net-ResNet18 and (**B**,**B’**): U-Net-ResNet34.

The loss and accuracy curves between different backbone learning frameworks are shown in Figure 2A,A′ for U-Net-ResNet18 and in Figure 2B,B′ for U-Net-ResNet34. Each model was configured to train for up to 200 epochs. However, early stopping was used to prevent overfitting with patience of 5 while monitoring the test accuracy. Specifically, if there was no improvement in test accuracy for five consecutive epochs, the training process was halted automatically. This strategy ensured that the model did not continue learning non-generalizable patterns. To further stabilize and enhance the training process, batch normalization layers and increased dropout were incorporated into the architecture. As illustrated in Figure 2A,A′, as well as in Figure 2B,B′, both showed a decrease in training and validation losses, while train accuracy and test accuracy improved on both sets until approximately the 20th and 19th epochs, respectively. Here, the test set represents the validation set. Given the limited dataset, the models are learning very fast with a very limited number of epochs and generalizing well on the unseen dataset. Early stopping was triggered around this point, and the best-performing weights were saved, helping to maintain optimal performance and minimize the risk of overfitting. We report the model test accuracy, precision, recall, F-1 score, and IoU (Intersection over Union) in Table 1. The results show that the ResNet-18-based U-Net architecture achieved a better segmentation test performance, with 90.74% accuracy, 75.98% precision, 79.57% recall, 77.74% F-1 score, and 63.58% IoU. It is also demonstrated that more informative biofilm features are learned through a deeper encoder architecture.

It provides a balance between learning biofilm features and maintaining the generalization of the test samples. When comparing deeper architectures, such as U-Net-ResNet34, it performed with improved precision, but the recall was low. This says that it struggles to capture all relevant biofilm regions, leading to under-segmentation. Additionally, DeeplabV3+ with multi-scale feature extraction through convolutions gives good accuracy and recall, but the precision is low, stating that it may misclassify non-biofilm regions targeting false positives. Additionally, Attention U-Net focuses on segmentation through attention mechanics; it has better recall, indicating that the detection of biofilm is high, and due to its lower precision and IoU scores, it tends to include non-biofilms regions, potentially causing over-segmentation. Lastly, the full CNN and BASNet performed well in terms of accuracy, but the models struggled with low precision, recall, and IoU.

**Figure 3.**
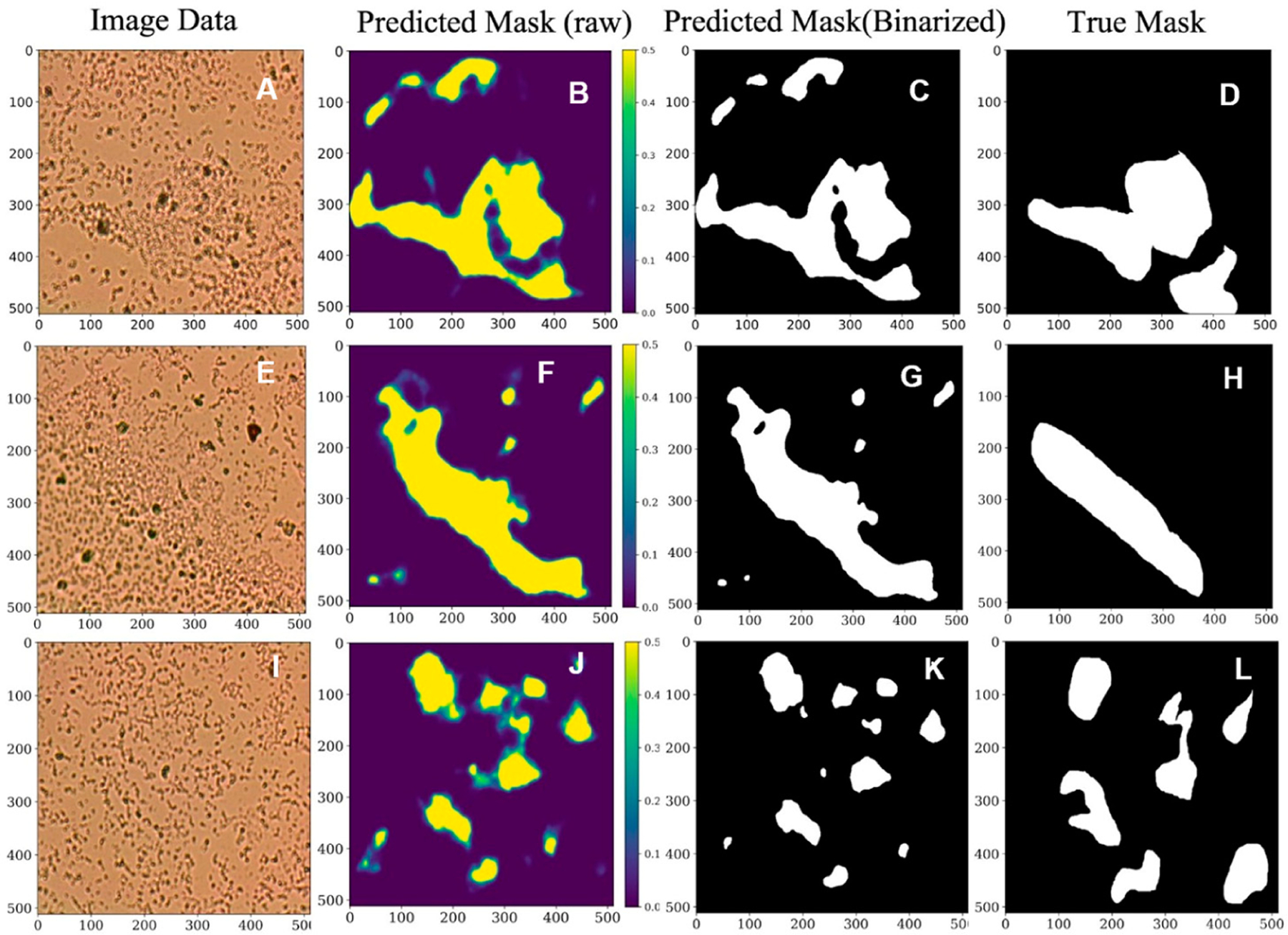
A test comparison of segmentation model raw output, predicted mask, and ground truth mask for different micrographs. Based on the image input (1st column (**A**,**E**,**I**)), the U-Net-ResNet18 algorithm generates the biofilm probability map (2nd column (**B**,**F**,**J**)). Otsu thresholding is applied to binarize the biofilm probability map to generate the predicted mask (3rd column (**C**,**G**,**K**)). The fourth column (**D**,**H**,**L**) shows the true mask provided by human experts as the comparison with the 3rd column.

Post-processing was performed to generate binary-predicted masks based on the output-predicted probability maps. Mainly, Otsu thresholding [45] was applied to binarize the segmentation prediction. A comparison of the model raw output mask and ground truth mask is shown in Figure 3; the first column has three different biofilm samples of *Pseudomonas aeruginosa* (PA) (Figure 3A,E,I). Figure 3D,H,L illustrate the related true masks provided by human experts. Note that Figure 3I is an image of *Pseudomonas aeruginosa* (PA) cells in a planktonic state. Based on each image input, the U-Net-ResNet34 algorithm, as shown in Figure 3, generates the related biofilm probability map, as shown in Figure 3B,F,J, where yellow shows the probability of being biofilm. Otsu thresholding is applied to binarize the biofilm probability map (Figure 3B,F,J) to generate the predicted mask, as shown in Figure 3C,G,K. The outperformed U-Net-ResNet34 architecture predicts the masks. It is demonstrated that the expected biofilm regions are aligned with the ground truth annotations, and the general shape of the biofilm is indicated. Hence, the ResNet-based U-Net method has great potential in this studied biofilm segmentation task. The experimental results demonstrated that the proposed model could achieve effective segmentation performance by generating accurate biofilm predictions compared to ground truth masks. Further quantitative measures can be computed and analyzed with this prediction.

## 3. Experimental Methodology and Instrumentation

### 3.1. Preparation of DNA-Templated Silver Nanocluster

The silver nanocluster (Ag-NC) was prepared on the aptamer DNA 5′-CCC CCG TTG CTT TCG CTT TTC CTT TCG CTT TTG TTC GTT TCG TCC CTG CTT CCT TTC TTG-3′ (which is specific for PA [46]), following the protocol published elsewhere [47].

This DNA oligonucleotide was custom-synthesized from Integrated DNA Technologies (IDT, Coralville, IA, USA). Lyophilized DNA was hydrated with triple distilled water (obtained from Sigma, Tokyo, Japan). Ag-NC was synthesized by combining 15 μM DNA and 90 μM Ag^+^ with ${6\text{BH}}_{4}^{-}$/oligonucleotide solutions, followed by vortex mixing for 1 min. The sample was stored overnight in the dark at 4 °C. UV/V is absorption, and fluorescence emission studies were conducted in the solution.

### 3.2. Preparation of Bacterial Samples for Biofilm Study

*Pseudomonas aeruginosa* ATCC 10145, Lot 416-116-4, was obtained from Microbiologics Inc., St. Cloud, MN, USA. PA was grown in 200 mL Tryptic Soy Broth (TSB, FisherSci., Hampton, NH, USA) at 23 °C overnight, and 25% of Ag-NC solution was added to media containing bacterial culture. In a separate study, PA in 100% media and in the presence of 25% water (the same volume added for Ag-NC) showed little difference in bacterial growth. Hence, this work has used PA culture with 25% water as a control. The plates were incubated at 23 °C for two days in six-well plates. Following our previous work, turbidity in the wells indicated biofilm formation [7,48]. Cells from the plates were heat-fixed on the glass slides for bright-field imaging.

### 3.3. Steady-State Absorption, Fluorescence

We performed steady-state absorption and fluorescence spectroscopic measurements to confirm Ag-NC formations. Steady-state absorption spectra were recorded with a Shimadzu UV 2550 spectrophotometer (Kyoto, Japan). Steady-state fluorescence measurements were carried out with a PerkinElmer FL 6500 fluorescence spectrophotometer (Shelton, CT, USA). Excitation and emission slit widths were 5/10 nm. All reported luminescence spectra were corrected for the detector′s spectral response.

The fluorescence and absorption experiments were performed to characterize the spectral properties of the DNA aptamer scaffolded silver nanoclusters, which were reported in our previous work [8]. We repeated these measurements to ensure the reproducibility of the Ag-NC formation on the DNA-templated aptamer and were able to reproduce similar results to those reported in [8]. The repeated measured fluorescence and absorption spectra have been reported in the Appendix A for the completeness of this article. In brief, for example, fluorescence emission spectra of aptamer-DNA 5′-CCC CCG TTG CTT TCG CTT TTC CTT TCG CTT TTG TTC GTT TCG TCC CTG CTT CCT TTC TTG-3′ templated Ag-NC show the absorption band around the wavelength region 380–390 nm. An absorption band peaking around 427 nm, with a shorter band peaking around ~530 nm, was observed. This indicates the formation of more than one type of silver nanocluster. Furthermore, the emission spectra of Ag-NCs showed stronger fluorescence with $\lambda_{em}^{max}$ at ~633 nm for *λ*_*ex*_ =540 nm, compared to the emission band with $\lambda_{em}^{max}$ at 530 nm for *λ*_*ex*_ = 450 nm, agreeing with our earlier work [8].

### 3.4. Instrumentation

**Figure 4.**
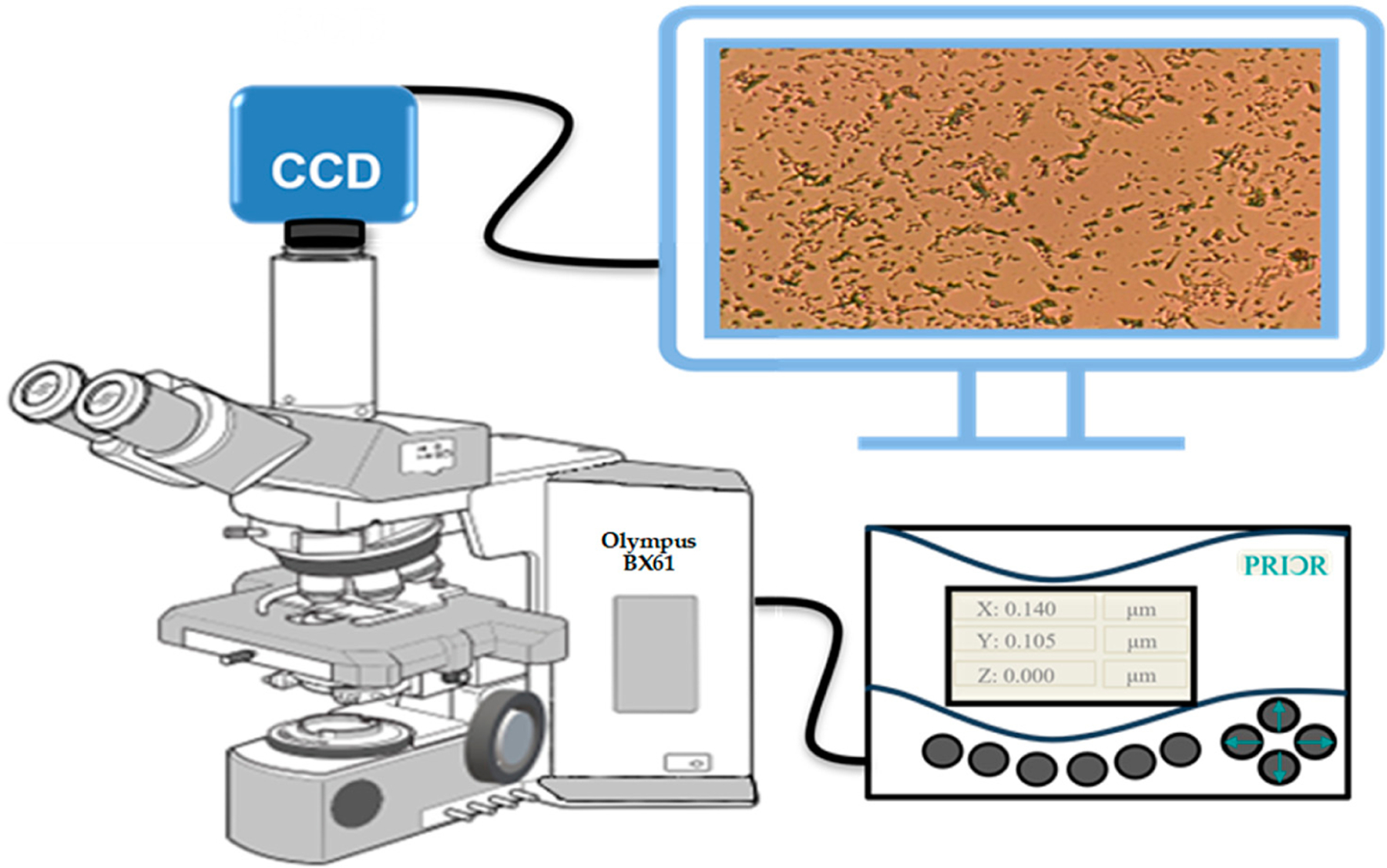
Schematic picture of a large-volume bright-field imaging system using an Olympus BX61 microscope (this picture is based on the Olympus manual guide) and Prior automated control system. The slide was kept on the holder of the x-y-z automated microscope stage and then scanned by a programmable matrix array. The scan speed is around ~1000 spot/hr.

Olympus BX61 optical microscope (Tokyo, Japan), CCD camera, and PRIOR Test Control (OptiScan software, PriorTest version 2.24.0.0) were used for large-volume bright-field transmission imaging. Rather than the traditional way of manual imaging, we used the autoscan feature of the microscope to identify and diagnose the biofilm prevention/formation and their statistical mixtures in thin biofilms on glass slides. To image the biofilms, we used a conventional Olympus BX61 (Figure 4) motorized system microscope with a 40X objective (UIS2) series, and a CCD camera mounted on the top of a BX61 microscope. In addition, we used the PRIOR Test Control via OptiScan software to move the microscope stage to synchronize with the camera while taking the scattering micrographs in the transmission mode.

### 3.5. Scanning Method

The most significant squares within each observation site of the biofilm over the glass slide were determined and divided into numbers proportional to the objective lens used (40X). Altogether, ~2000 microscopic micrographs were generated from each type of sample (C, CN1, CN2, CN3…) for the AI analyses.

### 3.6. AI: ResNet-Based U-Net Biofilm Segmentation

**Figure 5.**
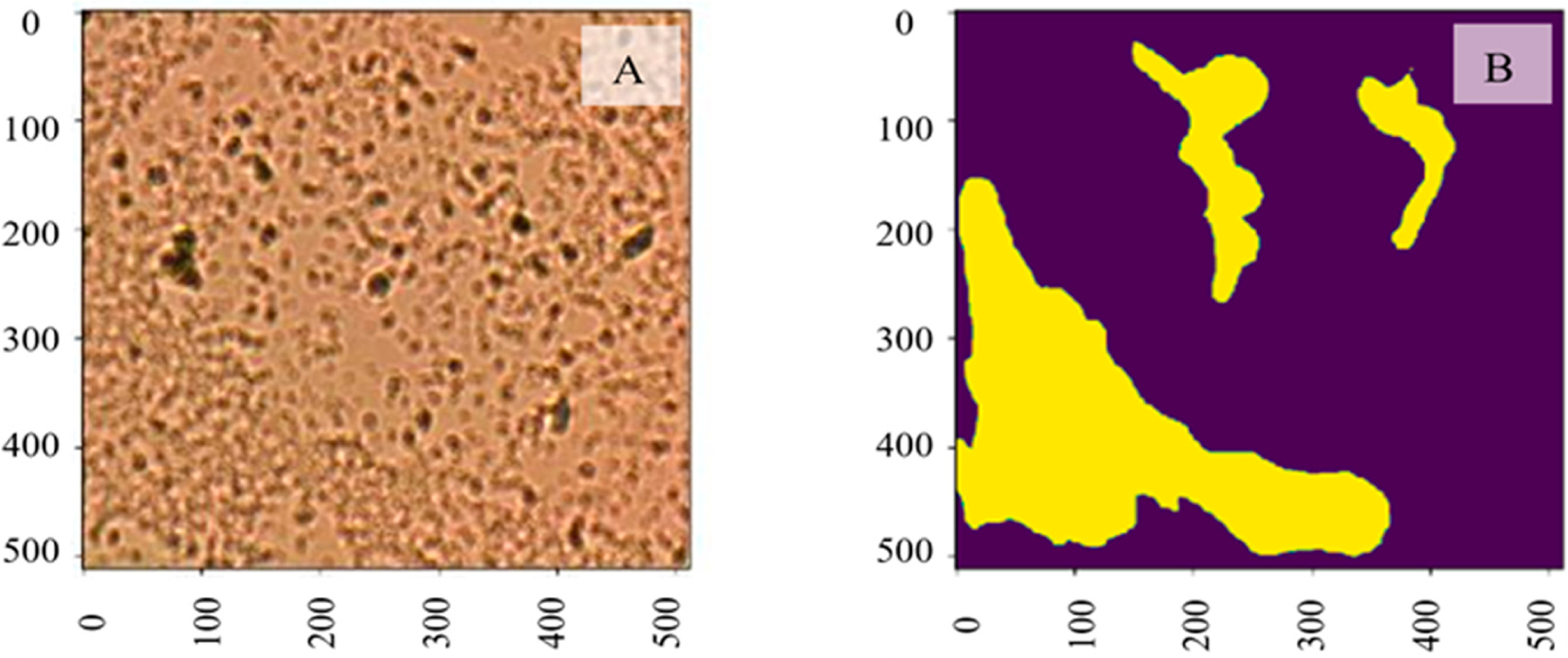
(**A**) Input image data captured by the bright-field imaging system; (**B**) the corresponding manually annotated mask (yellow areas indicate biofilm).

In this section, a U-Net-based segmentation model is used to segment biofilm to further retrieve the demanded quantitative measures of the bacteria, such as the covered area and cell count. We performed a pixel-level biofilm semantic segmentation using a U-Net1-based [40,49] AI framework within the studied regions. The analyzed dataset includes a total of 184 microbial images with annotated biofilm masks. Data robustness is ensured by tolerating variations in its collection and annotation procedure, where one image sample may contain from none to multiple segmented biofilm regions. The dataset is divided into a training set with ~150 samples and a test set with 34 samples. Each image datum with annotated masks is resized to the same input shape 512 × 512. The pixel values are normalized between 0 and 1. A preprocessed image sample with its ground truth mask is shown in Figures 5A and 5B, respectively. The bright-field imaging system captured the input image data, and domain experts collected and validated the corresponding manually annotated masks (yellow areas indicate biofilm).

UNet is widely recognized as an effective AI model for biomedical image segmentation. The network architecture contains a fully convolutional encoder-decoder with skip connections between the encoder blocks and their symmetric decoder blocks. This encoder-decoder structure of U-Net has inspired many segmentations in medical imaging; for instance, the attention mechanism has been employed in medical image segmentation and become widely adopted. The variation of U-Net-related deep learning networks is designed to optimize results by improving medical image segmentation′s accuracy and computing efficiency through changing network structures.

The convolution scheme is modified and extended in the conventional U-Net framework to work with few training images and produce more accurate segmentation. The general shrinkage network is replaced with sequential layers. The high resolution of the contracted path is combined with the upsampled output for localization. Therefore, sequential convolutional layers can study informative features and output more accurate segmentation. The network applies the practical part of every convolution, where the segmentation map contains mere pixels, and the complete context of the pixels can be obtained in the input image.

**Figure 6.**
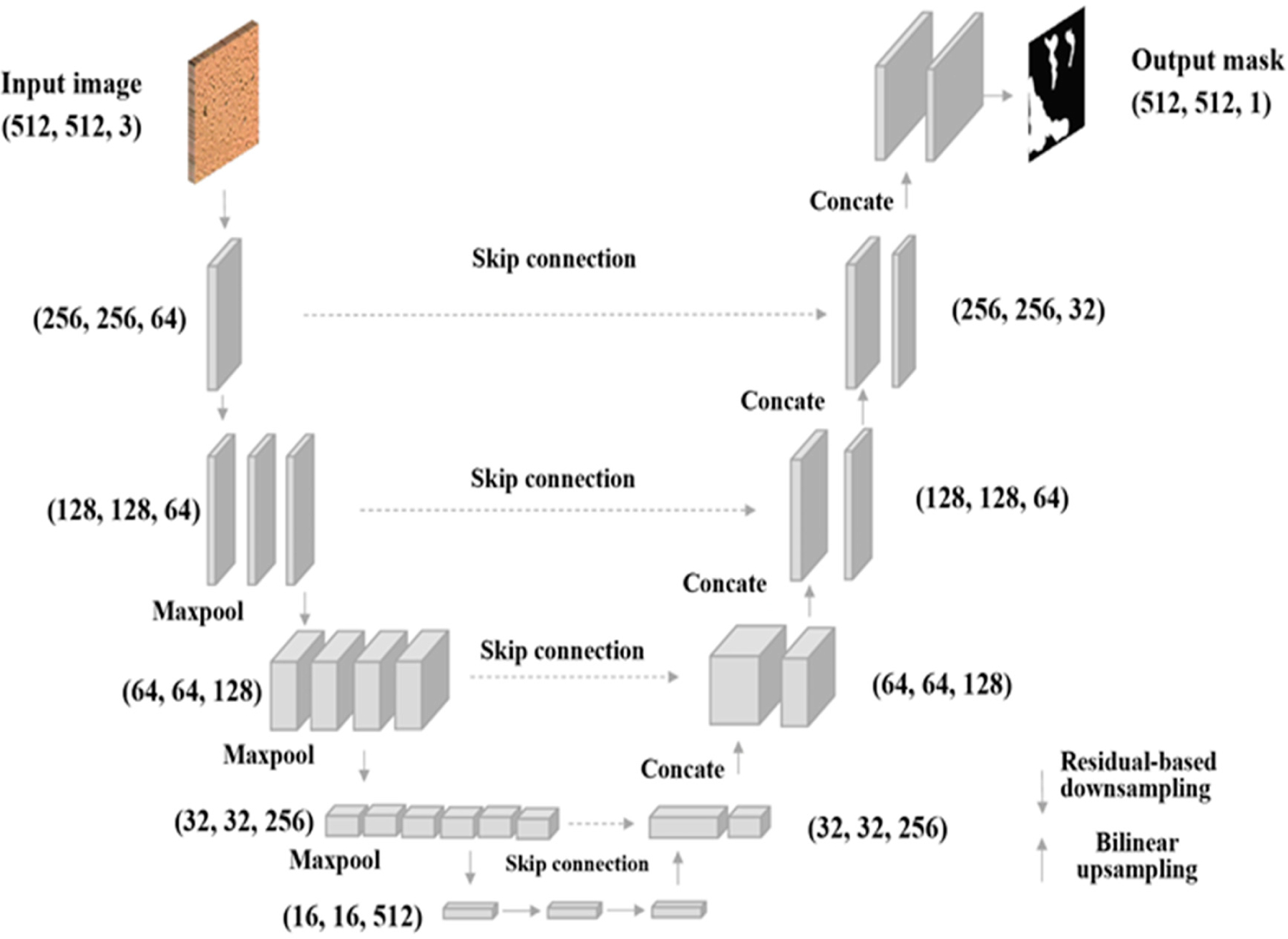
Architecture of the U-Net model. The model takes the input with image size 512 × 512 × 3. The downsampling process performs the feature extraction and compression, and up sampling performs the segmentation map generation. The output of the model is the biofilm probability map.

ResNet [37–39] introduces skip connections, allowing the network to skip specific layers to let the model learn effectively. A U-Net framework built with a ResNet encoder can leverage the power of deep residual learning and enhance the feature extraction process to improve the overall segmentation performance [41]. In addition, the shortcut mechanism added by the ResNet tends to avoid gradient vanishing and improve the network convergence efficiency. Because biofilm is even more complex for humans to identify with visual information, extensive and essential features related to biofilm are expected to be captured through a deep network learning process. The proposed ResNet-based U-Net segmentation framework is shown in Figure 6. This study investigated U-Net architecture with ResNet18 and ResNet34 backbones, respectively.

## 4. Conclusions and Discussion

We experimented to prevent biofilm formation using silver nanoclusters, which were synthesized on DNA aptamer matrices and were used to treat the bacterial biofilms of *Pseudomonas aeruginosa*. The experimental results showed that in the presence of Ag-NC, the degree of the 2D biofilm formation decreased.

The biofilm formation/prevention analysis was performed using AI-based models. We have performed the accuracy of the model using several standard-AI models: DeepLabV3+F-CN, BASNet, Attention U-net, U-Net-ResNet34, and U-Net-ResNet18. Our results show that U-Net-Resnet18 and U-Net-Resnet34 have the top accuracy.

The AI algorithm was trained for two cases: the formation and prevention of biofilm, followed by the testing of the AI model for its ability to detect biofilm among many images of *Pseudomonas aeruginosa*. The results show that the accuracy of detection is 91%. The developed technique can be used to quickly detect biofilms by healthcare, biotech, and environmental agencies. In our study, we currently employ early stopping and model check-pointing to save the best-performing weights based on validation performance. Additional techniques, such as data augmentation (e.g., rotation, scaling, and elastic deformations) and dropout regularization, could be incorporated to improve generalization further. In addition, transfer learning with pre-trained encoders, self-supervised learning, and synthetic data augmentation using generative models (e.g., GANs or diffusion models) could enhance model performance in our low-data settings, which is one of our potential future directions.

## Figures and Tables

**Table 1.** Test performance comparison of the studied AI models.

Model	Accuracy	Precision	Recall	F-1 Score	IoU
DeepLabV3+	0.9042	0.7491	0.7949	0.7713	0.6278
F-CN	0.8160	0.7554	0.8160	0.7338	0.6661
BASNet	0.8160	0.6659	0.8160	0.7333	0.6659
Attention U-net	0.8636	0.6138	0.8867	0.7254	0.5691
U-Net-ResNet34	0.8840	0.6766	0.8220	0.7422	0.5901
U-Net-ResNet18	0.9074	0.7598	0.7957	0.7774	0.6358

**Figure A1.**
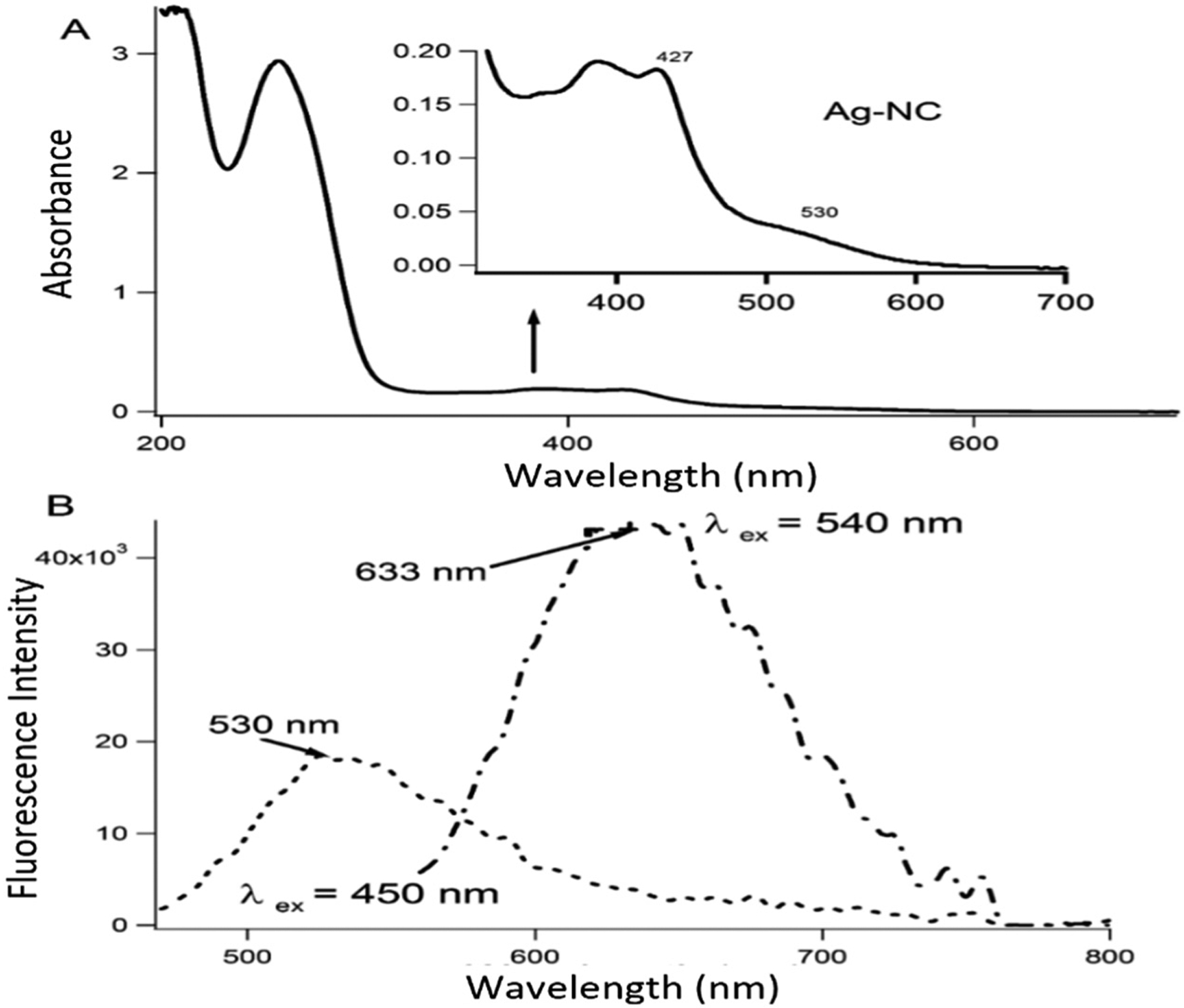
(**A**,**B**) shows typical absorption and fluorescence spectra of aptamer-DNA templated Ag-NC. The inset in Figure (**A**) focuses on the Ag-NC′s absorption region only.

## Data Availability

Data are available from the corresponding author (P.P.) on personal request.

## Appendix A. Characterization of the Aptamer-Templated Ag-Nc Using the Absorption and Fluorescence Spectroscopy

The absorption and fluorescence spectroscopic characterization of the Ag-NCs in aptamer has been reported in our earlier publication using a series of DNA aptamers [8]. We re-experimented the most efficient targeted aptamer [8] for forming Ag-NC and verifying the repeatability of our previous work. The results show similar absorption and fluorescence emission profile features to our earlier publication [8], indicating the Ag-NC formation on the aptamer template.

Figure A1 shows the typical absorption and fluorescence emission spectra of aptamer-DNA 5′-CCC CCG TTG CTT TCG CTT TTC CTT TCG CTT TTG TTC GTT TCG TCC CTG CTT CCT TTC TTG-3′ templated Ag-NC. The Ag-NCs were made in deionized water with DNA (15 μM), AgNO_3_ (90 μM), and NaBH_4_ (90 μM) to optimize nanoparticle formation, which is shown by the absorption band around the 380–390 nm region. The absorption of the nucleobases in the UV region is shown in Figure A1, while the inset highlights the absorbance of Ag-NC. An absorption band with a peak around 427 nm and a shorter band of ~530 nm were observed, proving the formation of more than one type of silver nanocluster on this PA-specific aptamer template, which agrees with our previous work [8]. The emission profiles of these Ag-NCs show that for *λ*_***ex***_ =540 nm, the NC showed stronger fluorescence with $\lambda_{em}^{max}$ at ~633 nm compared to the emission band with $\lambda_{em}^{max}$ at 530 nm for *λ*_***ex***_= 450 nm, agreeing with our earlier work [8].
